# Hydronephrosis in patients with cervical cancer is an indicator of poor outcome: A nationwide population-based retrospective cohort study

**DOI:** 10.1097/MD.0000000000024182

**Published:** 2021-02-12

**Authors:** You-Rong Yang, Szu-Ju Chen, Pin-Yeh Yen, Chi-Ping Huang, Lu-Ting Chiu, Wu-Chou Lin, Huey-Yi Chen, Yung-Hsiang Chen, Wen-Chi Chen

**Affiliations:** a Department of Urology, Department of Obstetrics & Gynecology, Management Office for Health Data, China Medical University Hospital; b Division of Urology, Department of Surgery, Taichung Veterans General Hospital; c Graduate Institute of Integrated Medicine, College of Chinese Medicine, China Medical University; d Department of Psychology, College of Medical and Health Science, Asia University, Taichung, Taiwan.

**Keywords:** cervical cancer, hydronephrosis, nationwide population-based study, ureteral catheter

## Abstract

Cervical cancer is a common malignancy in women. The presence of hydronephrosis in patients with cervical cancer can be a challenging clinical problem. The appropriate management of these patients and the prediction of their outcomes are concerns among gynecologists, urologists, medical oncologists, radiation oncologists, and nephrologists. We enrolled a total of 2225 patients with cervical cancer over a 12-year period from the nationwide database of Taiwan's National Health Insurance Bureau. Among them, 445 patients had concomitant hydronephrosis. The remaining 1780 patients without hydronephrosis were randomly enrolled as a control group for the analysis of associated factors. The results indicated that the proportions of patients with hypertension, chronic kidney disease, and diabetes were significantly higher in the hydronephrosis group. The hydronephrosis group showed a higher all-cause mortality than the non-hydronephrosis group (adjusted hazard ratio 3.05, 95% confidence interval 2.24–4.15, *P* < .001). The rates of nephrectomy and stone disease were also significantly higher in the hydronephrosis group. A higher percentage of other cancers was also observed in the hydronephrosis group than in the non-hydronephrosis group (12.36% vs 8.99%, respectively). This study shows that cervical cancer with hydronephrosis may have a higher morbidity and mortality than cervical cancer without hydronephrosis. Other factors such as human papilloma virus vaccination, smoking, and cancer staging need to be further studied.

## 1 Introduction

Cervical cancer is the fourth most common malignancy in women in Taiwan.^[1–3]^ In total, 4285 patients were reported to have carcinoma in situ and invasive cervical cancer in 2018. The treatment of cervical cancer depends on the cancer stage, cancer type, patient's age, associated clinical conditions, and patient's desire to bear children. The clinical condition of hydronephrosis represents an advanced disease in patients with cervical cancer, as it indicates the involvement of the parametria. Patel et al analyzed 279 patients with cervical cancer and found that hydronephrosis involved more advanced cancer stage at any time point.^[4]^ It may result in pain, infection, and deterioration of renal function caused by obstruction. Ureteral stent placement is always the first-choice initial treatment by urologists for relieving the obstruction.^[4]^ However, percutaneous nephrostomy (PCN) is an alternative when ureteral stenting is not feasible or ineffective.

In 2010, Rose et al demonstrated that relief of ureteral obstruction is correlated with improved outcome in patients with cervical cancer.^[5]^ However, further studies have shown that ureteral stent placement is associated with a poor prognosis in patients with gynecologic malignancies. A systematic review recently pointed out that there is still a lack of consensus on the management of these patients.^[6]^ Although hydronephrosis is considered to be associated with a poor prognosis,^[7]^ it remains unclear whether ureteral stent placement is beneficial to these patients. In view of the lack of understanding of the timing of hydronephrosis development, the outcome of patients with cervical cancer associated with hydronephrosis remains unclear. Therefore, in this study, we aimed to investigate this issue by using a nationwide database to study the timing of hydronephrosis development during the course of cervical cancer, and to analyze the outcome and comorbidities of these patients.

## 2 Methods

### 2.1 Data source

In 1995, Taiwan launched National Health Insurance (NHI) program, which is a compulsory and single-payer program that currently covers nearly the total population. This study was conducted using the LHID2000, which is a subset from NHIRD that comprises the data of 1,000,000 randomly sampled beneficiaries of the NHI program. This database contains extensive inpatient and outpatient data, including demographic characteristics, information of disease, medical treatment and prescription medications. All diseases are diagnosed based on the International Classification of Diseases, Ninth Revision, Clinical Modification (ICD-9) codes. Personal data were scrambled into electronic format for public access, and detail information of the program were noted in previous studies. This study has been approved by the Research Ethics Committee at China Medical University Hospital (CMUH104-REC2-115- CR-4).

### 2.2 Subject selection

**Figure 1:**
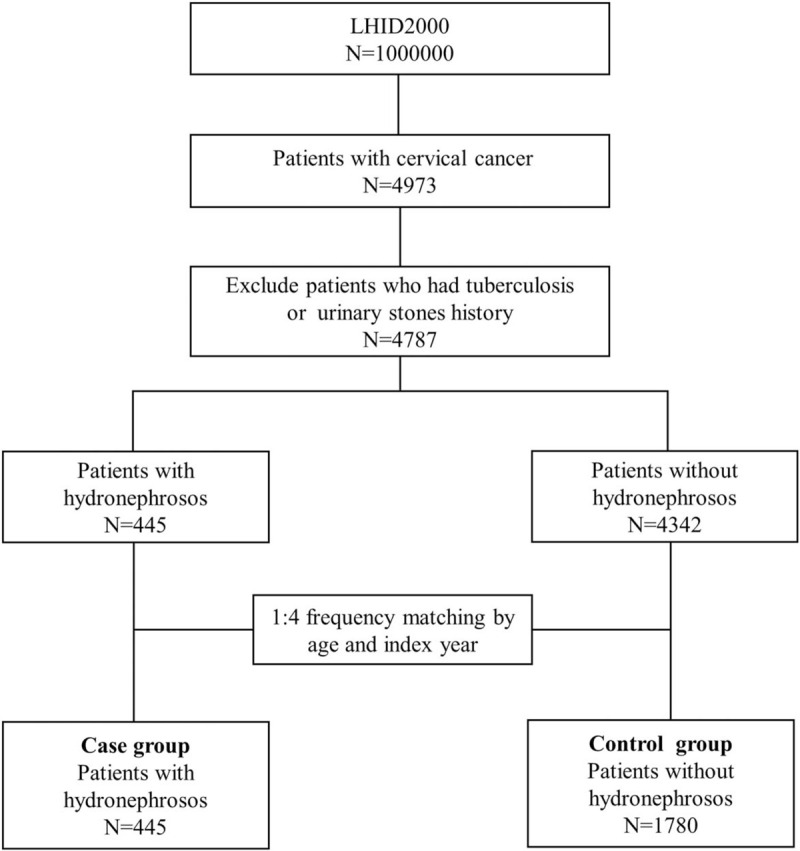
Flow chart shows the enrolment of the participants in the study cohort.

The target population of this population-based retrospective cohort study were female patients who aged above 18 and with cervical cancer (ICD-9-CM code 180) diagnosed by gynecologist from 2000 to 2012. We then identified subjects who diagnosed with hydronephrosis (ICD-9-CM code 591) after cervical cancer as the case group. The diagnosis date of hydronephrosis was defined as the index date. Subjects without a diagnosis of hydronephrosis were randomly selected from the target population as the comparison group. The cases and the matched comparison group were frequency matched according to age (every 5 years), and the year of the index date. The ratio of case group and comparison group was 1:4. Patients who had chronic kidney disease (ICD-9-CM code 582, 583, 585, 586 and 588), tuberculosis (ICD-9-CM code 010-018) or urinary stones (ICD-9-CM code 592.1, 594.1, 592.0 and 592.9) history before the index date were excluded from the study. A total of 2225 subjects were included in this study (Fig. 1).

### 2.3 Outcome and comorbidities

The outcomes of interest in this study were listed as following: all-cause mortality, urethra catheter (ICD-9-CM procedures code 59.8, 56.0), chronic kidney disease, double J (DJ) removal (ICD-9-CM procedures code 57.32), PCN (ICD-9-CM procedures code 55.03), keep DJ (ICD-9-CM treatment code 77023), infection (ICD-9-CM code 590.10, 599.0), urinary stones, diversion (ICD-9-CM treatment code 78014B, 78045, 78046B, 78042B, 78041B and 78012B) and nephrectomy (ICD-9-CM procedures code 55.5). All study patients were censored at the earliest date of outcome identified, withdrawal from the insurance program, or the end of 2013. We calculated the follow-up time of each outcome event. Related comorbidities considered for each patient were diabetes mellitus (ICD-9-CM code 250), hypertension (ICD-9-CM code 401-405), chronic kidney disease (ICD-9-CM code 585) and cancer (ICD-9-CM code 140-208). The status of chronic kidney disease was identified during the follow-up period, while other comorbidities were identified at baseline.

### 2.4 Statistical analyses

Statistical analysis in the first-stage analysis, we examined differences between the hydronephrosis patients and the comparison group for age and comorbidities, using the Chi- square and t tests. In the main analysis, the hazard ratio (HR) and accompanying 95% confidence interval (CI) were estimated by univariable and multivariable Cox proportional hazard regression models to analyze the incidences of outcomes between the hydronephrosis cases and the comparison group. The multivariable model was controlled for age, gender and comorbidities. In the subsequent analysis, we stratified the time of hydronephrosis development after cervical cancer was diagnosed (<1 week, 1 week–6 month, >6 month) for the following outcome: all-cause mortality, urethra catheter, chronic kidney disease, DJ removal, PCN, keep DJ, infection, and urinary stones. All statistical analyses were performed using SAS statistical software, version 9.4 (SAS Institute, Inc., Cary, NC, USA). A 2 tailed *P* value less than .05 was considered to indicate a statistical significance.

## 3 Results

The distributions of age and comorbidities are shown in Table 1. In total, we categorized 445 cervical cancer patients with hydronephrosis into the case group (hydronephrosis group) and 1780 cervical cancer patients without hydronephrosis into the comparison group (non-hydronephrosis group). After matching, no age differences were observed between the 2 groups. The mean age of the study subjects was about 59 years. The hydronephrosis group had significantly higher proportions of patients with diabetes and chronic kidney disease than the non-hydronephrosis group. With respect to previous cancers as a comorbidity, 70 (3.93%) patients in the non-hydronephrosis group and 18 (4.04%) patients in the hydronephrosis group were associated with other cancers, and the difference between the groups was not statistically significant. A further analysis of other cancers associated with cervical cancer is shown in Table 4.

**Table 1:** Characteristics of hydronephrosis group and non-hydronephrosis group among patients with cervical cancer.

	Hydronephrosis				
	No (n = 1780)		Yes (n = 445)		
Characteristics	n	%	n	%	*P* value
Age (yr)					1.00
18–50	520	29.21	130	29.21	
50–65	644	36.18	161	36.18	
>65	616	34.61	154	34.61	
Mean ± SD	58.67 ± 14.10	58.78 ± 14.03	0.88		
Comorbidities					
Diabetes	322	18.09	111	24.94	.001
Hypertension	676	37.98	191	42.92	.05
CKD	152	8.54	125	28.09	<.0001
Previous cancer	70	3.93	18	4.04	.91
Follow-up (yr)	5.80 ± 4.81	4.70 ± 4.18	<.0001		

Data shown as n (%) or mean ± SD. CKD = chronic kidney disease; the status of CKD was defined as the occurrence during follow-up period, exclude tuberculosis (all study period), CKD and urinary stones history (before index date), using 1:4 frequency matching based on age and index year. ^∗^*P* < .05 ^∗∗^*P* < .01 ^∗∗∗^*P* < .001.

The mean follow-up period of the hydronephrosis and non-hydronephrosis groups was 4.70 � 4.18 and 5.80 � 4.81 years, respectively. The Cox proportional hazard regression models for analyzing the person-years, incidence, and hazard ratio of outcome risks contributing to hydronephrosis are shown in Table 2.

**Table 2:** Outcome comparisons between hydronephrosis group and non-hydronephrosis group in patients with cervical cancer.

	Non-hydronephrosis (n = 1780)			hydronephrosis (n = 445)			Crude HR	Adjust HR
Outcomes	Events	PY	IR	Events	PY	IR	(95% CI)	(95% CI)
All-cause mortality	104	11806	8.81	79	2185	36.16	3.96 (2.95–5.31)^∗∗∗^	3.05 (2.24–4.15)^∗∗∗^
Urethral catheter	164	11507	14.25	113	2069	54.62	4.86 (3.53–6.69)^∗∗∗^	3.87 (2.77–5.41)^∗∗∗^
CKD	152	11462	13.26	125	1870	66.84	4.73 (3.73–6.00)^∗∗∗^	2.60 (2.02–3.33)^∗∗∗^
Removal of DJ	61	11587	5.26	70	1919	36.48	3.16 (2.23–4.48)^∗∗∗^	2.94 (2.01–4.29)^∗∗∗^
Referred for PCN	7	11775	0.59	102	1927	52.93	33.78 (15.57–73.30)^∗∗∗^	33.06 (14.81–73.77)^∗∗∗^
Keep DJ	7	11768	0.59	133	1642	81.00	31.42 (14.61–67.58)^∗∗∗^	30.36 (13.89–66.36)^∗∗∗^
Infection	384	9793	39.21	116	1568	73.98	1.14 (0.92–1.40)	1.22 (0.98–1.51)
Urinary stones	92	11347	8.11	95	1671	56.85	1.98 (1.48–2.65)^∗∗∗^	1.90^∗^1.41–2.56)^∗∗∗^
Diversion	11	11758	0.94	1	2185	0.46	0.62 (0.08–5.97)	0.71 (0.04–10.51)
Nephrectomy	52	11568	4.50	15	2101	7.14	2.02 (1.13–3.59)^∗∗∗^	2.03 (1.09–3.76)^∗∗∗^

HR adjusted for age, gender, diabetes mellitus, hypertension, chronic kidney disease (CKD). CI = confidence interval, HR = hazard ratio, IR = incidence rate, per 1000 person-years, PCN = percutaneous nephrostomy, PY = person-years.

∗ *P* < .05.

∗∗ *P* < .01.

∗∗∗ *P* < .001.

After adjusting for potential confounders, the hydronephrosis group was at a significantly higher risk for all-cause mortality (hazard ratio [HR] 3.05, 95% confidence interval [CI] 2.24–4.15), urethral catheter insertion (HR 3.87, 95% CI 2.77–5.41), chronic kidney disease (HR 2.60, 95% CI 2.02–3.33), double J (DJ) stent removal (HR 2.94, 95% CI 2.01–4.29), PCN (HR 33.06, 95% CI 14.81–73.77), retained DJ stent (HR 30.36, 95% CI 13.89–66.36), urinary stones (HR 1.90, 95% CI 1.41–2.56), and nephrectomy (HR 2.03, 95% CI 1.09–3.76) than the non-hydronephrosis group. Stone disease was present in 92 (5.16%) and 95 (21.34%) patients in the non-hydronephrosis and hydronephrosis groups, respectively.

To investigate whether the length of time for developing hydronephrosis would affect the risk of outcomes, we performed a subsequent analysis. The results are shown in Table 3. After stratification by follow-up time, the multivariable model showed no significant association between outcome risks and the timing of hydronephrosis development.

**Table 3:** Risk of outcomes among cervical cancer patients with hydronephrosis stratified by developed timing after cervical cancer diagnosed.

Developed timing	Event	PY	IR	Crude HR (95% CI)	Adjusted HR (95% CI)
All-cause mortality (n = 79)					
<1 week	17	740	22.97	0.59 (0.34–1.03)	0.76 (0.39–1.51)
1 week–6 month	12	399	30.08	0.72 (0.38–1.37)	0.82 (0.43–1.56)
>6 month	50	1045	47.85	1 (reference)	1 (reference)
Urethral catheter (n = 72)					
<1 week	17	681	24.96	0.70 (0.40–1.24)	0.84 (0.41–1.73)
1 week–6 month	12	366	32.79	0.85 (0.44–1.63)	1.01 (0.52–1.94)
>6 month	43	1021	42.12	1 (reference)	1 (reference)
CKD (n = 125)					
<1 week	33	610	54.10	0.80 (0.53–1.21)	1.08 (0.61–1.92)
1 week–6 month	14	373	37.53	0.52 (0.29–0.92)	0.68 (0.37–1.26)
>6 month	78	886	88.04	1 (reference)	1 (reference)
Removal of DJ (n = 70)					
<1 week	25	622	40.19	2.33 (1.40–3.88)^∗∗^	2.14 (1.08–4.22)
1 week–6 month	8	353	22.66	0.69 (0.32–1.49)	0.82 (0.37–1.82)
>6 month	37	944	39.19	1 (reference)	1 (reference)
PCN (n = 102)					
<1 week	24	680	35.29	0.57 (0.34–0.94)^∗^	0.69 (0.35–1.36)
1 week–6 month	19	325	58.46	0.80 (0.47–1.34)	0.82 (0.48–1.40)
>6 month	59	921	64.06	1 (reference)	1 (reference)
Keep DJ (n = 133)					
<1 week	26	610	42.62	1.17 (0.75–1.84)	1.20 (0.66–2.17)
1 week–6 month	32	228	140.35	1.04 (0.68–1.57)	1.14 (0.75–1.76)
>6 month	75	804	93.28	1 (reference)	1 (reference)
Infection (n = 116)					
<1 week	39	493	79.11	1.52 (1.01–2.30)^∗^	1.16 (0.66–2.06)
1 week–6 month	21	274	76.64	1.26 (0.76–2.09)	1.26 (0.75–2.11)
>6 month	56	800	70.00	1 (reference)	1 (reference)
Urinary stones (n = 95)					
<1 week	27	551	49.00	0.95 (0.60–1.51)	1.10 (0.59–2.07)
1 week–6 month	11	344	31.98	0.76 (0.93–1.46)	0.74 (0.38–1.45)
>6 month	57	774	73.64	1 (reference)	1 (reference)

HR adjusted for age, gender, diabetes mellitus, hypertension, chronic kidney disease (CKD) and cancer. CI = confidence interval, HR = hazard ratio, IR = incidence rate, per 1000 person-years, PCN = percutaneous nephrostomy, PY = person-years.

∗ *P* < .05.

∗∗ *P* < .01. ^∗∗∗^*P* < .001.

Table 4 lists the other cancers associated with cervical cancer in both groups. In the non-hydronephrosis group, 70 patients (3.93%) had previous cancers before cervical cancer. In the hydronephrosis group, 18 patients (4.04%) had previous cancers. Gynecologic, colon, and breast cancers were the 3 most commonly seen malignancies in both groups. After the cervical cancer diagnosis, 119 (6.69%) and 43 (9.66%) other cancers were diagnosed in the non-hydronephrosis and hydronephrosis groups, respectively. In the non-hydronephrosis group, the commonly seen cancers were the same as the cancers diagnosed before cervical cancer, whereas gynecologic cancer was the most frequently seen cancer in the hydronephrosis group (19 in 43 patients). In total, 160 patients (8.99%) in the non-hydronephrosis group and 55 patients (12.36%) in the hydronephrosis group were associated with other cancers, and the difference between groups was statistically significant (*P* = .03).

**Table 4:** Distribution of cancer type between renal edema group and non-renal edema group among patients with cervical cancer.

	Hydronephrosis				
	No (n = 1780)		Yes (n = 445)		
Cancer type	n	%	n	%	*P* value
All cancers	160	8.99	55	12.36	.03
Before cervical cancer					
All cancers	70	3.93	18	4.04	
Head and neck	3	0.17	0	0	
Esophagus	1	0.06	0	0	
Stomach	2	0.11	1	0.22	
Small intestine	0	0	0	0	
Colon	16	0.90	4	0.9	
Liver	3	0	0	0	
Pancreas	0	0	0	0	
Lung	3	0.17	0	0	
Skin	0	0	0	0	
Breast	13	0.73	3	0.67	
GYN cancer	22	1.24	5	1.12	
Bladder	3	0.17	4	0.9	
Kidney	1	0.06	1	0.22	
Hematological	3	0.17	0	0	
After cervical cancer					
All cancers	119	6.69	43	9.66	
Head and neck	5	0.28	2	0.45	
Esophagus	3	0.17	0	0	
Stomach	5	0.28	2	0.45	
Small intestine	1	0.06	0	0	
Colon	18	1.01	4	0.90	
Liver	12	0.67	3	0.67	
Pancreas	1	0.06	1	0.22	
Lung	10	0.56	1	0.22	
Skin	0	0	0	0	
Breast	16	0.90	2	0.45	
GYN cancer	32	1.8	19	4.27	
Bladder	6	0.34	4	0.90	
Kidney	5	0.28	4	0.90	
Hematological	9	0.51	2	0.45	

Data shown as n (%) or mean ± SD.

## 4 Discussion

This is the first study to focus on the timing of hydronephrosis and the comorbidities of patients with cervical cancer. The hydronephrosis group had significantly higher proportions of patients with diabetes, chronic kidney disease, urinary stones, nephrectomy, and other cancers in this study. Although the association between hydronephrosis and ureteral stricture is unclear, previous studies have suggested that diabetes can promote both tumorigenesis and tumor progression; thus, diabetes is a risk factor for cancer.^[8–10]^ Diabetes is one of the most common risk factors for chronic kidney disease and end-stage renal disease in Taiwan.^[11]^ The presence of several coexisting comorbidities in the hydronephrosis group may be the cause of high all-cause mortality in this group.

Ureteral obstruction is a major cause of kidney injury in patients with gynecologic cancer. Hydronephrosis or nonfunctioning kidney (unless known to be due to another cause) is assigned a stage of International Federation of Gynecology and Obstetrics (FIGO) IIIB by the FIGO staging of cancer of the cervix uteri (2018) and older versions.^[12,13]^ A single-institution case series reported that patients with <20% renal function may recover from acute kidney injury after the placement of a stent or PCN tube.^[14]^ Rose et al demonstrated that hydronephrosis is a significant but not independent prognostic factor, and relief of obstruction may improve the outcome of patients with stage IIIB cancer and disease restricted to the pelvis.^[5]^ However, Yoon et al reported that internal ureteral stenting was effective for maintaining, but not for restoring, renal function in patients with malignant ureteral obstruction.^[15]^ Our results showed that nearly two-thirds of patients had stenting or percutaneous drainage for the relief of obstruction. Furthermore, we found that although placement of a DJ stent may improve or maintain renal function, the prognosis seemed poor in these patients. The database used in this study did not provide staging of cervical cancer. Nevertheless, the presence of hydronephrosis indicated a more advanced cancer stage and more morbidities, resulting in higher mortality in this study. Further study may turn out to be investigating Stage IIIB vis-à-vis other stages, particularly lower-stage disease.

It is challenging to distinguish between morbidity from hydronephrosis and procedure-related morbidity. Stent change through cystoscopy is a less invasive procedure that is accompanied by a low probability of complications; therefore, it is preferred both by patients and physicians.^[16,17]^ This situation was also observed in our study. The rate of referral for PCN was low. Many patients with a ureteral stent experience frequent urinary tract infection and decreased renal function,^[17–19]^ which increase the risk of urolithiasis and the indications for nephrectomy. Meanwhile, ureteral obstruction may compromise the ability to deliver potentially nephrotoxic cisplatin chemotherapy concurrently with radiation therapy, leading to poor prognosis.^[8]^

Multiple primary cancers can be seen in 2% to 17% of patients.^[20]^ There are many underlying factors related to multiple cancers,^[21,22]^ such as lifestyle, viral infection, genetic factors, and treatment-related factors.^[23]^ Our study revealed that patients with cervical cancer with hydronephrosis had a higher proportion of other cancers (12.36% in total) than those without hydronephrosis (8.99% in total). The incidence of other cancers before cervical cancer was similar in the 2 groups. However, the incidence of other cancers that occurred after cervical cancer were higher in the hydronephrosis group than in the non-hydronephrosis group. Arnold et al screened patients with cervical cancer in the Netherlands cancer registry, and found that 5.6% were diagnosed with secondary cancers.^[24]^ They also found that the secondary cancers were mostly related to smoking, especially in patients treated with radiotherapy. Secondary cancer development after radiation treatment for cervical cancer was also reported by Boice et al.^[25]^ The status of hydronephrosis was not detected in their study. However, our database did not provide information about smoking. The relationship among smoking, hydronephrosis, and multiple cancers needs further studies.

As early as 2005, Ganatra et al published an algorithm for the management of extrinsic malignant ureteral obstruction.^[19]^ They claimed that the indications of referral for PCN were increased creatinine, worsening of hydronephrosis, flank pain, infection, or inability to replace the ureteral stent during the replacement procedure. The failure rate of ureteral stent placement was reported to be 16% to 58%.^[19,26]^ In our results, these problems may be avoided through more frequent stent changes, prophylactic antibiotic therapy, choosing a DJ stent made of a different material, and even antegrade DJ replacement. However, when and how to stent require further studies.

Hydronephrosis occurs at any time point in the course of cervical cancer, and the causes vary.^[27]^ Patel et al demonstrated that patients with cervical cancer who developed hydronephrosis at any time point showed poor survival.^[4]^ Theoretically, different timings and causes of hydronephrosis may lead to distinct outcomes. However, similar to Patel et al, we observed that hydronephrosis occurring at any point in the course of cervical cancer leads to poor outcomes. This indicates that it is crucial to recognize and manage these comorbidities, as they are major threats to cervical cancer patients, even more than the malignancy itself.

Human papilloma virus (HPV) infection is a well-known high-risk factor for cervical cancer.^[23]^ HPV vaccination has been reported to be effective in preventing cervical cancer.^[28]^ Comprehensive vaccination for HPV during puberty has been initiated in 2018 in Taiwan. The effect on prevention was not well documented. Our database did not provide information about the patients’ self-vaccination behaviors. Therefore, this study lacked this information.

The proportion of patients with stone disease in the hydronephrosis group was higher than that in the non-hydronephrosis group. More than one in 5 patients developed stone disease in the hydronephrosis group, which was predictable owing to a high percentage of patients who received DJ stent indwelling. A DJ stent may act as a foreign body in the urinary tract and may be a nidus of stone formation.^[29]^ A high infection rate (>70%) may be another cause of stone formation, which is related to infectious stones.^[30]^

The limitations of this retrospective study included the limited number of patients, lack of biochemical data and cancer staging, small patient number in subgroup analysis of the timing of hydronephrosis development to reach significance, and faults inherent in a retrospective analysis. The database was limited to 12-year records. No records of HPV vaccination was available in this database for a further analysis of its effect

## 5 Conclusions

Hydronephrosis in patients with cervical cancer, regardless of the timing of development, was highly associated with morbidities such as diabetes, chronic kidney disease, urinary stones, nephrectomy, and other cancers, which may be the cause of poor prognosis and high all-cause mortality. This is the first study to focus on the timing of hydronephrosis and the comorbidities of patients with cervical cancer. A future prospective study should enroll a larger number of patients and compare the progression, complications, and quality of life between patients managed with PCN tubes and retrograde stenting.

## Acknowledgments

We also thank China Medical University and Hospital for supporting this study (DMR-110-073 and CMU109-S-37).

## Author contributions

**Conceptualization:** You-Rong Yang, Szu-Ju Chen, Lu-Ting Chiu, Wu-Chou Lin, Huey-Yi Chen, Wen-Chi Chen.

**Data curation:** You-Rong Yang, Szu-Ju Chen, Pin-Yeh Yen, Chi-Ping Huang.

**Formal analysis:** You-Rong Yang, Szu-Ju Chen, Chi-Ping Huang, Lu-Ting Chiu, Wu-Chou Lin, Huey-Yi Chen.

**Funding acquisition:** Yung-Hsiang Chen, Wen-Chi Chen.

**Investigation:** You-Rong Yang, Szu-Ju Chen, Pin-Yeh Yen, Chi-Ping Huang, Lu-Ting Chiu, Wu-Chou Lin, Huey-Yi Chen, Yung-Hsiang Chen, Wen-Chi Chen.

**Methodology:** You-Rong Yang, Pin-Yeh Yen, Chi-Ping Huang, Wen-Chi Chen.

**Project administration:** Wen-Chi Chen.

**Resources:** Wen-Chi Chen.

**Software:** You-Rong Yang, Szu-Ju Chen.

**Supervision:** Wen-Chi Chen.

**Validation:** Szu-Ju Chen, Pin-Yeh Yen, Chi-Ping Huang, Lu-Ting Chiu, Wu-Chou Lin, Huey-Yi Chen, Yung-Hsiang Chen, Wen-Chi Chen.

**Writing – original draft:** You-Rong Yang, Szu-Ju Chen, Yung-Hsiang Chen, Wen-Chi Chen.

**Writing – review & editing:** Yung-Hsiang Chen, Wen-Chi Chen.

## Abbreviations

Abbreviations: CI = confidence intervals, DJ = double J, FIGO = International Federation of Gynecology and Obstetrics, HPV = human papilloma virus, HR = hazard ratio, ICD-9-CM = International Classification of Diseases, Ninth Revision, Clinical Modification, LHID = Longitudinal Health Insurance Database, NHI = National Health Insurance, NHIRD = National Health Insurance Research Database, PCN = percutaneous nephrostomy.
